# Chikungunya Fever Outbreak, Zhejiang Province, China, 2017

**DOI:** 10.3201/eid2508.181212

**Published:** 2019-08

**Authors:** Junhang Pan, Chunfu Fang, Juying Yan, Hao Yan, Bingdong Zhan, Yi Sun, Ying Liu, Haiyan Mao, Guoping Cao, Lei Lv, Yanjun Zhang, Enfu Chen

**Affiliations:** Zhejiang Provincial Center for Disease Control and Prevention, Hangzhou, China (J. Pan, J. Yan, H. Yan, Y. Sun, Y. Liu, H. Mao, Y. Zhang, E. Chen);; Quzhou Center for Disease Control and Prevention, Quzhou, China (C. Fang, B. Zhan, G. Cao, L. Lv)

**Keywords:** chikungunya, outbreak, China, viruses, chikungunya virus

## Abstract

We report a disease outbreak caused by chikungunya virus in Zhejiang Province, China, in August 2017. Phylogenic analysis indicated that this virus belonged to the Indian Ocean clade of the East/Central/South African genotype and was imported by a traveler returning from Bangladesh.

Chikungunya fever is an arboviral disease transmitted between humans and through the bites of infected *Aedes* mosquitoes, specifically the species *Ae. aegypti* and *Ae. albopictus* (*1*). High fever, myalgia, polyarthralgia, and maculopapular rash are typical clinical symptoms of chikungunya fever. However, some chikungunya virus (CHIKV) infections have led to severe clinical symptoms, such as neurologic signs or fulminant hepatitis, which have had a serious effect on human health (*2*).

CHIKV was isolated in 1952 during a dengue outbreak in Tanzania; a CHIKV outbreak in Asia was reported in Thailand in 1960. Since 2004, CHIKV has caused unexpected large outbreaks in Africa, Asia, and the Americas, becoming a major public health concern throughout the world (*3*). A nonindigenous case of CHIKV infection in mainland China was reported in 1986; no outbreaks resulting from local transmission were reported until the chikungunya outbreaks in Dongguan and Yangjiang regions in Guangdong Province, in southern China, in 2010 (*4*). Three imported cases of CHIKV infection were confirmed in travelers returning from Southeast Asia in Zhejiang Province, eastern China: 2 cases in 2008 and 1 in 2012.

Zhejiang Province is located in eastern China (27°01′–31°10′N, 118°01′–123°08′Ε) and has a humid subtropical monsoon climate. Its average annual temperature is 15°C–18°C, and it has abundant rainfall, an average of 1,100–2,200 mm annually. The mosquito vector *Ae. aegypti* has not been found in Zhejiang Province, whereas the range of *Ae. albopictus* mosquitoes was distributed around this region (*5*).

Zhejiang Province does not belong to the endemic area of chikungunya; however, we report an outbreak of chikungunya caused by a traveler returning from Bangladesh in August 2017 in Quzhou, Zhejiang Province. The patient with the imported case was living in Huangdun village; another 3 CHIKV infections were confirmed in the same village (Appendix Figure). After the confirmation of these infections, the Quzhou government quickly organized mosquito control measures: granules of fenthion were used to control the mosquito larvae, and the wettable powders of cyfluthrin and cyhalothrin were used to control the adult mosquito. The Breteau index (number of positive containers per 100 houses inspected) in Huangdun village decreased from 114 on August 23 to <20 in 2 days. No similar case was reported during the continuous monitoring in hospitals in Quzhou, and serum samples collected from the inhabitants of this village were negative for CHIKV RNA, IgM, and IgG.

We inoculated serum samples of these 4 cases on C6/36 cell lines to isolate CHIKV, and we observed complete cytopathic effects on all incubations. CHIKV RNA was confirmed in these samples; we named these strains ZJQZ3, ZJQZ4, ZJQZ5, and ZJQZ6. Sequence analysis of the CHIKV envelope (E) 1 gene showed that 4 sequences were 100% identical with one another, which indicated that the infected traveler who returned from a disease-endemic area led to this local transmission and outbreak. We sequenced the complete genome sequence of CHIKV ZJQZ3 (11,765 bp; GenBank accession no. MG912993) using Ion Torrent PGM (https://www.thermofisher.com*)* and performed phylogenetic analysis with 45 worldwide CHIKV strain sequences from GenBank. Results showed that the genome sequence of CHIKV ZJQZ3 was highly similar to the strain (GenBank accession no. MF773566) isolated from a patient in Australia who returned from Bangladesh in 2017 and belongs to the Indian Ocean clade of the East/Central/South African genotype (Figure). However, a large outbreak of chikungunya fever was observed in Dhaka, Bangladesh, during May–August 2017 (*6*). With an increase in global travel, the risk for spreading CHIKV to the regions in which the virus is not endemic has increased.

**Figure:**
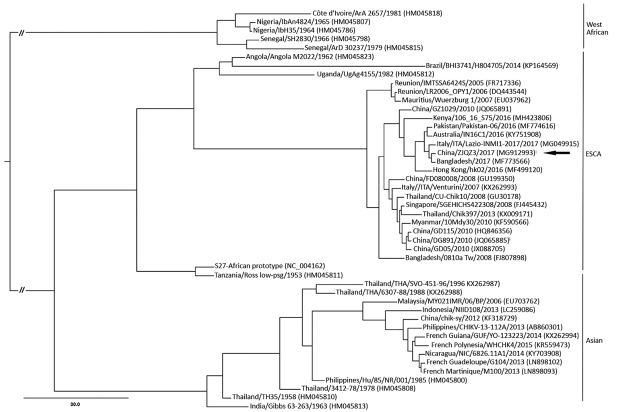
Phylogenetic analysis of the complete CHIKV genome sequences of isolate ZJQZ3 from Quzhou, Zhejiang Province, China (arrow), and reference sequences. Dataset-specific models that were selected using the Akaike Information Criterion in Modeltest 3.7 (http://darwin.uvigo.es/our-software) were analyzed. Maximum-likelihood (ML) analysis was processed in RAxML v7.2.8 (http://sco.h-its.org/exelixis/software.html). The optimal ML tree and bootstrap percentages (BP) were estimated in the same run. The ML BP values were obtained from 1,000 bootstrap replicates using the rapid bootstrap algorithm. BEAST 1.6 (http://beast.community/programs) was employed to construct a Bayesian maximum clade credibility tree based on an uncorrelated exponential distributed relaxed-clock model for our sample. The genotypes of CHIKV were divided into West African, ECSA, and Asian. Virus lineages are shown at right. GenBank accession numbers are given in parentheses. Scale bar indicates nucleotide substitutions per site. CHIKV, chikungunya virus; ECSA, East/Central/South African.

We observed mutations in E1-M269V, E1-D284E, and nonstructural (ns) gene P3-D372E in CHIKV ZJQZ3 but found no *Ae. albopictus*–adaptive mutations in E1-A226V, E2-L210Q, E2-K252Q, E2-K233E, and E2/E3-R198Q/S18F (*7*–*9*). However, the mutations in E1-A226V, E2-K252Q, E2-L210Q, and E2-V264A were reported previously in some imported cases in China (*10*). The growing genetic diversities observed in each strain suggested that CHIKV will be a major public health threat with the potential for further emergence and spread.

This outbreak indicates that CHIKV can be transmitted by *Ae. albopictus* mosquitoes in Zhejiang Province and may have the potential for further emergence and spread in northern China. Clinicians should be educated about the diagnosis of this disease, and public health organizations should work to overcome the diagnostic challenges of multiple arboviruses, carefully monitor imported cases, strengthen vector control, and conduct surveillance for CHIKV-infected vectors in high-risk areas to prevent local establishment of this new emerging virus.

Appendix Location of the sites of chikungunya virus infection in Quzhou, Zhejiang Province, China.

## References

[1] Burt FJ, Chen W, Miner JJ, Lenschow DJ, Merits A, Schnettler E, et al. Chikungunya virus: an update on the biology and pathogenesis of this emerging pathogen. Lancet Infect Dis. 2017;17:e107–17. 28159534 10.1016/S1473-3099(16)30385-1

[2] Rolph MS, Foo SS, Mahalingam S. Emergent chikungunya virus and arthritis in the Americas. Lancet Infect Dis. 2015;15:1007–8. 10.1016/S1473-3099(15)00231-5 26333330

[3] Burt FJ, Rolph MS, Rulli NE, Mahalingam S, Heise MT. Chikungunya: a re-emerging virus. Lancet. 2012;379:662–71. 10.1016/S0140-6736(11)60281-X 22100854

[4] Wu D, Wu J, Zhang Q, Zhong H, Ke C, Deng X, et al. Chikungunya outbreak in Guangdong Province, China, 2010. Emerg Infect Dis. 2012;18:493–5. 10.3201/eid1803.110034 22377135 PMC3309566

[5] Yang T, Fu G. Investigation on the distribution of dengue vector *Aedes albopictus* in Zhejiang Province. Chinese Journal of Hygienic Insecticides and Equipments. 2006;12:189–91.

[6] Kabir I, Dhimal M, Müller R, Banik S, Haque U. The 2017 Dhaka chikungunya outbreak. Lancet Infect Dis. 2017;17:1118. 10.1016/S1473-3099(17)30564-9 29115257

[7] Tsetsarkin KA, Vanlandingham DL, McGee CE, Higgs S. A single mutation in chikungunya virus affects vector specificity and epidemic potential. PLoS Pathog. 2007;3:e201. 10.1371/journal.ppat.0030201 18069894 PMC2134949

[8] Tsetsarkin KA, Weaver SC. Sequential adaptive mutations enhance efficient vector switching by Chikungunya virus and its epidemic emergence. PLoS Pathog. 2011;7:e1002412. 10.1371/journal.ppat.1002412 22174678 PMC3234230

[9] Tsetsarkin KA, Chen R, Yun R, Rossi SL, Plante KS, Guerbois M, et al. Multi-peaked adaptive landscape for chikungunya virus evolution predicts continued fitness optimization in *Aedes albopictus* mosquitoes. Nat Commun. 2014;5:4084. 10.1038/ncomms5084 24933611 PMC7091890

[10] Zheng K, Li J, Zhang Q, Liang M, Li C, Lin M, et al. Genetic analysis of chikungunya viruses imported to mainland China in 2008. Virol J. 2010;7:8. 10.1186/1743-422X-7-8 20078896 PMC2831882

