# Supplementary figures and images for: Chikungunya Fever Outbreak, Zhejiang Province, China, 2017
Source: Emerg Infect Dis. 2019 Aug;25(8):1589–91. doi: 10.3201/eid2508.181212 (PMC6649353; doi:10.3201/eid2508.181212)

# Chikungunya Fever Outbreak, Zhejiang Province, China, 2017

## Appendix

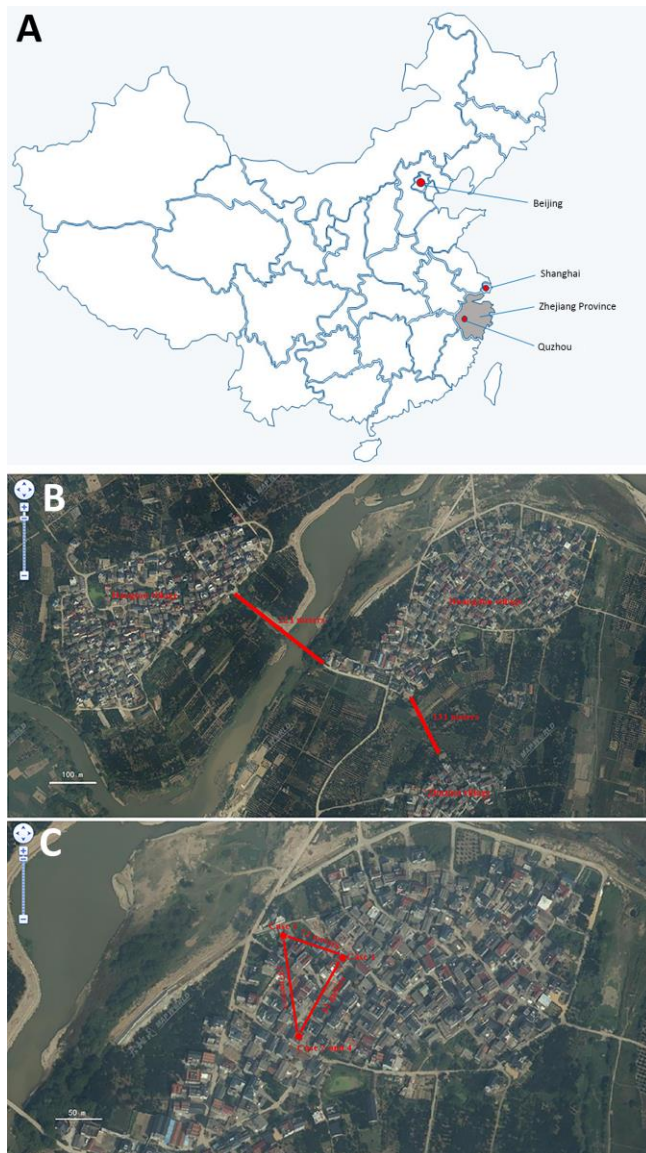

Supplement: Appendix — Location of the sites of chikungunya virus infection in Quzhou, Zhejiang Province, China. [file 18-1212-Techapp-s1.pdf]
